# Allelic expression analysis of the osteoarthritis susceptibility gene *COL11A1* in human joint tissues

**DOI:** 10.1186/1471-2474-14-85

**Published:** 2013-03-08

**Authors:** Emma V A Raine, Andrew W Dodd, Louise N Reynard, John Loughlin

**Affiliations:** 1 Newcastle University, Musculoskeletal Research Group, Institute of Cellular Medicine, 4th Floor Catherine Cookson Building, The Medical School, Framlington Place, Newcastle upon Tyne NE2 4HH, UK

**Keywords:** Osteoarthritis, Lumbar disc herniation, Genetics, Susceptibility, *COL11A1*, Allelic expression

## Abstract

**Background:**

The single nucleotide polymorphism (SNP) rs2615977 is associated with osteoarthritis (OA) and is located in intron 31 of *COL11A1*, a strong candidate gene for this degenerative musculoskeletal disease. Furthermore, the common non-synonymous *COL11A1* SNP rs1676486 is associated with another degenerative musculoskeletal disease, lumbar disc herniation (LDH). rs1676486 is a C-T transition mediating its affect on LDH susceptibility by modulating *COL11A1* expression. The risk T-allele of rs1676486 leads to reduced expression of the *COL11A1* transcript, a phenomenon known as allelic expression imbalance (AEI). We were keen therefore to assess whether the effect that rs1676486 has on *COL11A1* expression in LDH is also observed in OA and whether the rs2615977 association to OA also marked AEI.

**Methods:**

Using RNA from OA cartilage, we assessed whether either SNP correlated with *COL11A1* AEI by 1) measuring *COL11A1* expression and stratifying the data by genotype at each SNP; and 2) quantifying the mRNA transcribed from each allele of the two SNPs. We also assessed whether rs1676486 was associated with OA susceptibility using a case–control cohort of over 18,000 individuals.

**Results:**

We observed significant AEI at rs1676486 (p < 0.0001) with the T-allele correlating with reduced *COL11A1* expression. This corresponded with observations in LDH but the SNP was not associated with OA. We did not observe AEI at rs2615977.

**Conclusions:**

*COL11A1* is subject to AEI in OA cartilage. AEI at rs1676486 is a risk factor for LDH, but not for OA. These two diseases therefore share a common functional phenotype, namely AEI of *COL11A1*, but this appears to be a disease risk only in LDH. Other functional effects on *COL11A1* presumably account for the OA susceptibility that maps to this gene.

## Background

Primary osteoarthritis (OA) is a common disease of the synovial joint characterised by articular cartilage thinning and loss, which is often accompanied by alteration in the normal function of other tissues of the joint [1]. A number of epidemiological studies have demonstrated that osteoarthritis has a major genetic component, although elucidation of this has proven challenging [2].

Genome-wide association scans (GWASs) are now being employed as a powerful, objective tool for mapping OA susceptibility loci [3-6]. In some circumstances the resulting signals are to regions of the genome that do not harbour obvious OA candidates, whereas in others the signals are close to, or even within, a gene that would be considered a strong candidate. One example of the latter emerged from the original arcOGEN GWAS [4]. This GWAS and its subsequent replication analysis was performed on individuals of European descent and included the full arcOGEN samples and, amongst other signals, identified association to single nucleotide polymorphism (SNP) rs2615977, with a p-value of 1.2×10^-5^ and an odds ratio of 1.10 for the T-allele of the SNP.

rs2615977 resides within intron 31 of *COL11A1*. This gene codes for the α1 polypeptide chain of type XI collagen, a quantitatively minor but structurally important component of the cartilage extracellular matrix. Mutations within *COL11A1*, typically involving the substitution of highly conserved glycine residues or the deletion of whole exons, are responsible for certain forms of Stickler syndrome, a rare and usually dominant Mendelian condition that presents with a severe and premature form of OA manifesting during adolescence [7,8].

Lumbar disc herniation (LDH) and lumbar disc degeneration (LDD) can be viewed as having similar aetiological routes to OA, with each condition being age-associated and involving the degeneration of cartilage, albeit of different types, with OA being hyaline and LDH/LDD being fibrocartilage. The common non-synonymous SNP rs1676486, which is located in exon 62 of *COL11A1*, has been reported to associate with LDH in a Japanese population [9]. rs1676486 is a C-T transition that results in a proline to serine substitution. Using an *in vitro* assay the investigators were able to demonstrate that the LDH-associated T-allele of rs1676486 correlated with decreased stability of the *COL11A1* transcript relative to the C-allele. Such a difference in allelic expression, whether mediated by differential mRNA transcription or mRNA transcript stability, is known as allelic expression imbalance (AEI). As such, the investigators concluded that a quantitative deficiency of *COL11A1*, and by extrapolation a quantitative deficiency of the α1 polypeptide chain of type XI collagen, was a contributor to LDH susceptibility.

As more susceptibility loci are identified for common human diseases it is becoming clear that many, if not the majority, of associated alleles contribute to disease risk by influencing gene expression, typically by modulating rates of transcription or transcript stability [10,11]. The T-allele of rs1676486 is an example of such an effect in LDH. It is also becoming apparent that in most of these cases the effect that the susceptibility allele has on the transcript can be organ, tissue or even cell type-specific [12,13]. It is important therefore to study RNA extracted from tissues relevant to the disease of interest when assessing the potential effect of a functional susceptibility allele.

Based on these observations we hypothesised that the OA association to *COL11A1*, marked by rs2615977, may be mediated by modulating the expression of the gene in articular cartilage. In addition, we hypothesised that the effect that rs1676486 has on *COL11A1* expression in LDH may also be observed in OA. To investigate these hypotheses we have quantitatively measured overall expression of *COL11A1* in cartilage and then stratified our data by genotype at rs2615977 and at rs1676486. We have also tested for AEI of *COL11A1* using assays that can accurately discriminate and quantify the mRNA output from each allele of a transcript SNP.

## Methods

### Patients and tissues

Macroscopically normal articular cartilage tissue located away from the OA lesion was obtained from individuals undergoing elective joint replacement for OA of the hip (total hip replacement, THR) or of the knee (total knee replacement, TKR), as described in detail previously [14,15]. The Newcastle and North Tyneside research ethics committee granted ethical approval for the collection (REC reference number 09/H0906/72) and written informed consent was obtained from donors for the use of their tissue, and permission for publication of their age and sex. Details regarding the patients can be found in Additional file 1: Table S1 and Additional file 2: Table S2.

### Nucleic acid extraction

On the day of surgery, the tissue specimens were snap-frozen at −80°C. For each individual tissue sample, 0.5-1.0 g of frozen tissue was ground to a powder using a Retsch mixermill 200 (Retsch Limited, Leeds, UK) under liquid nitrogen, which causes the sample to become brittle and prevents the RNA from degrading. Genomic DNA and RNA were then extracted from the ground tissue samples using an EZNA DNA/RNA Isolation Kit and a protocol established for human tissue (Omega bio-tek, R6731-02). The nucleic acid was quantified using a NanoDrop ND-1000 Spectrophotometer (NanoDrop Technologies, Wilmington, USA).

### SNPs

We studied three *COL11A1* SNPs: the OA associated SNP rs2615977, which is located in intron 31; the LDH associated SNP rs1676486, which is located in exon 62; and SNP rs9659030, which is located in the 3^′^UTR (Table 1). rs9659030 was analysed for AEI in the absence of a *COL11A1* transcript SNP in high linkage disequilibrium (LD) with the OA SNP rs2615977. rs2615977 is 98.2 kb from rs1676486 and 110 kb from rs9659030.

**Table 1:** **The three *COL11A1 *SNPs studied in this report**

**SNP**	**Alleles**	**MAF**^**1**^	**Pairwise linkage disequilibrium**					
			**Relative to rs2615977**		**Relative to rs1676486**		**Relative to rs9659030**	
			**D**^**′**^	**r**^**2**^	**D**^**′**^	**r**^**2**^	**D**^**′**^	**r**^**2**^
rs2615977	T/G	0.239	-	-	0.58	0.03	0.39	0.08
rs1676486	C/T	0.202	0.58	0.03	-	-	0.33	0.09
rs9659030	A/G	0.173	0.39	0.08	0.33	0.09	-	-

^1^Minor allele frequency in Europeans (HapMap CEU).

### SNP genotyping

The three *COL11A1* SNPs were genotyped by restriction fragment length polymorphism (RFLP) analysis (patient genotypes are listed in Additional file 1: Table S1). The primer sequences and restriction enzymes used can be found in Additional file 3: Table S3.

### cDNA synthesis and quantitative real-time PCR

RNA extracted from cartilage was reverse transcribed using the SuperScript First-Strand cDNA synthesis kit (Invitrogen). Gene expression was measured by quantitative real-time PCR using PrimeTime Mini qPCR Assays (Integrated DNA Technologies, Iowa, USA) and an ABI PRISM 7900HT Sequence Detection System (Applied Biosystems). *COL11A1* expression was measured relative to the average expression of the housekeeping genes *HPRT1*, *GAPDH* and *18S*: for each cDNA sample three pipetting replicates we performed per gene and the nine housekeeper cycle threshold (Ct) values (3x3) were combined to derive the mean Ct which was used as the control to compare against *COL11A1*. Expression of *COL11A1* relative to the housekeepers was determined using the 2^-∆ Ct^ method. Mann–Whitney U and Kruskal-Wallis tests were performed to assess whether *COL11A1* expression relative to genotype at rs2615977 and genotype at rs1676486 differed significantly from the null. All primer and probe sequences can be found in Additional file 4: Table S4.

### AEI analysis

AEI was assessed using the transcript SNPs rs1676486 and rs9659030 and quantitative real time PCR genotyping assays, purchased from Applied Biosystems. These assays are standard real time assays except that they employ a probe (labeled with FAM or VIC) specific to each of the two alleles of a SNP. Real time PCR was carried out according to the manufacturer’s instructions. In brief, 10 ng of cDNA or 20 ng of DNA extracted from the cartilage was added to 5 μl of TaqMan Universal Master Mix II no UNG (Applied Biosystems) and 0.25 μl of 40 × TaqMan assay in a 10 μl reaction. The samples were then amplified on an ABI PRISM 7900HT Sequence Detection System. The reactions were performed with five pipetting replicates and the allelic ratios were calculated using the formula (2^-FAM Ct^)/(2^-VIC Ct^).

For each assay, the ratios between the amounts of each allele in every sample were calculated for genomic DNA and cDNA. For each sample the average allelic ratio for genomic DNA, which represents the 1:1 ratio, was then used to normalise the cDNA ratio to generate a corrected allelic ratio. To determine if there was an overall difference in expression between alleles for a particular tissue across all patients the mean allelic ratios for the patient cDNAs were compared to the mean allelic ratios for the patient genomic DNAs using a Mann–Whitney *U* test.

### Quality control checks on the AEI assays

The capacity for each assay to discriminate between SNP alleles was verified using a standard curve of artificially created allelic ratios using DNA from samples homozygous for the major and the minor alleles. To assess the amplification efficiency of each AEI probe (two per assay) a standard curve was produced by performing AEI on a serial dilution of 200 ng to 1.5635 ng of DNA from a heterozygous sample.

### Association of rs1676486 with OA

We analysed the LDH associated SNP rs1676486 for evidence of association to OA using data from the UK arcOGEN study. This is a GWAS performed for 485,491 autosomal SNPs and conducted on 7,410 cases with severe OA of the hip or the knee, 80% of whom had undergone total joint replacement, and on 11,009 publically available population controls [6].

## Results

### Quantitative expression of *COL11A1* in cartilage stratified by genotype at the OA and LDH associated SNPs

**Figure 1:**
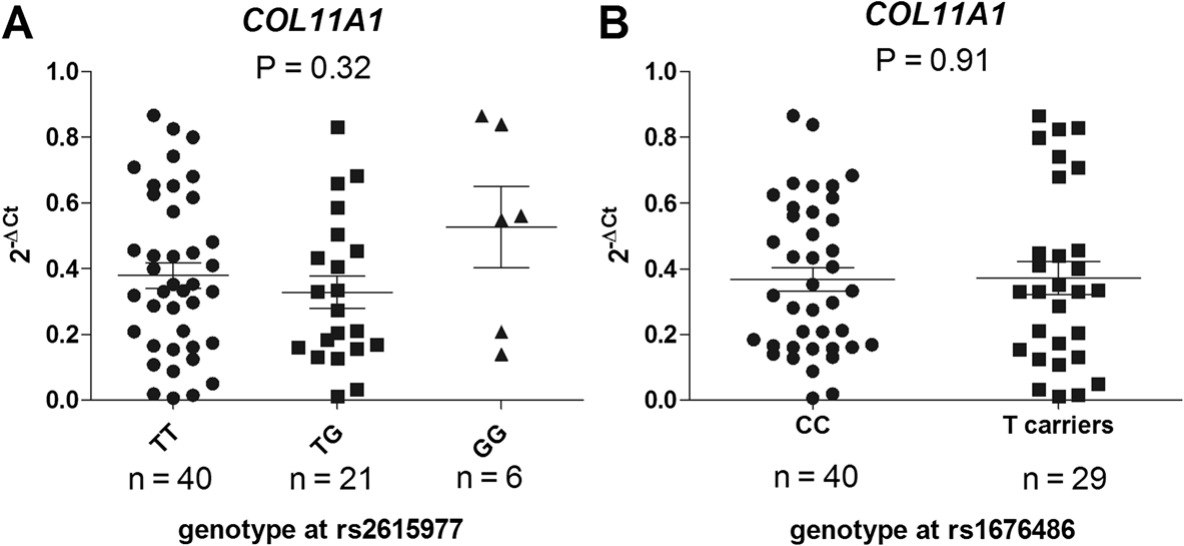
**Columnar scatter plot of the quantitative expression of *COL11A1 *in cartilage cDNA stratified by genotype at A) the OA associated SNP rs2615977 and B) the LDH associated SNP rs1676486.** Due to the low frequency of TT homozygotes at rs1676486 the analysis in **B**) was between CC homozygotes and T-allele carriers (CT and TT combined) at this SNP. n is the number of patients studied for each group. The horizontal lines in each plot represent the mean and the standard error of the mean. P-values were calculated using a Kruskal-Wallis test for **A**) and a Mann–Whitney *U* test for **B**).

**Figure 2:**
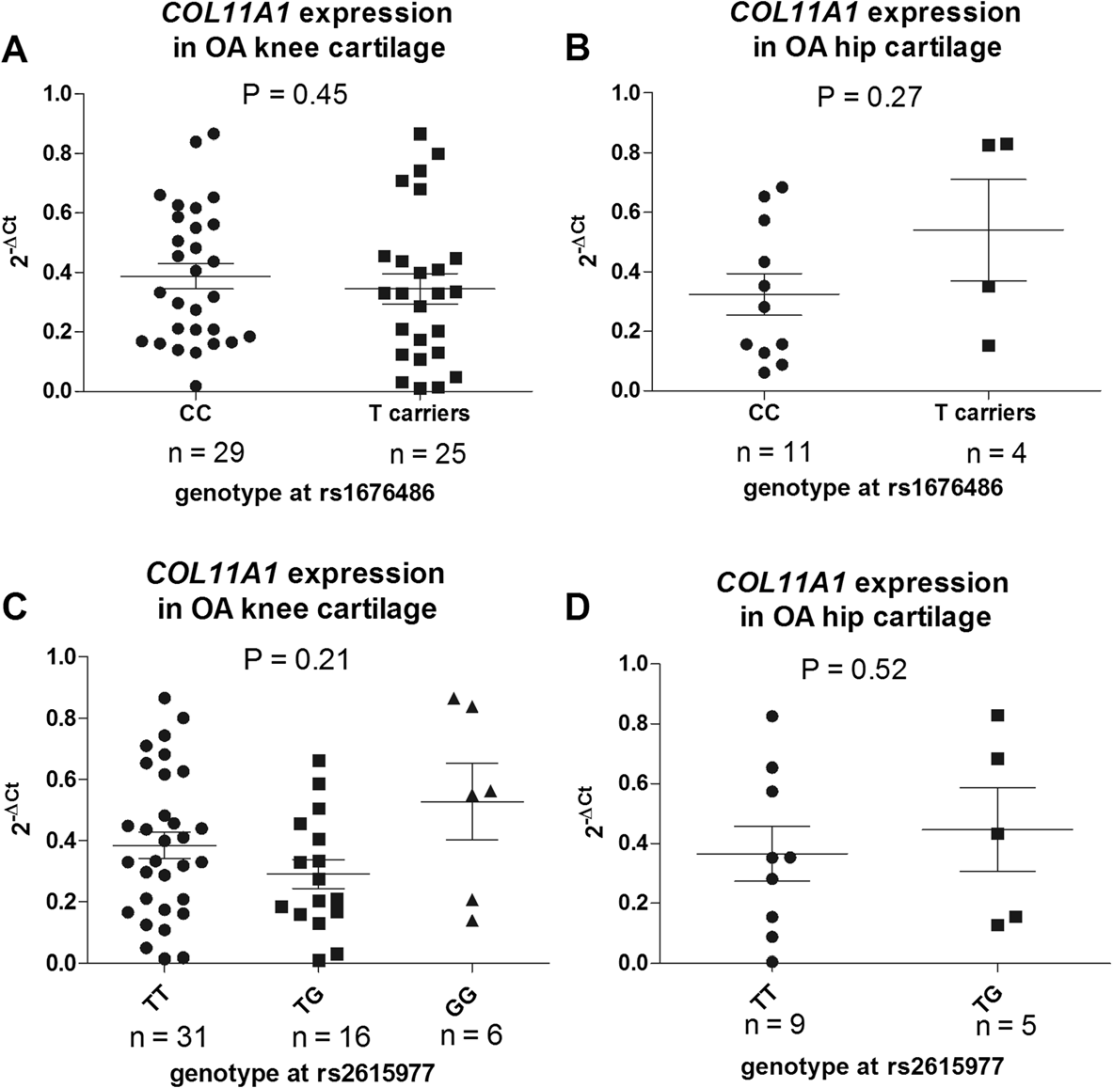
**Columnar scatter plots of the quantitative expression of *COL11A1 *in OA cartilage cDNA stratified by A) genotype at the LDH associated SNP rs1676486 in knee cases, B) genotype at the LDH associated SNP rs1676486 in hip cases, C) genotype at the OA associated SNP rs2615977 in knee cases, and D) genotype at the OA associated SNP rs2615977 in hip cases.** Due to the low frequency of TT homozygotes at rs1676486 the analysis in **A**) and **B**) was between CC homozygotes and T-allele carriers (CT and TT combined) at this SNP. Due to the absence in the hip strata of GG homozygotes at rs2615977 the analysis in **D**) was between TT homozygotes and TG heterozygotes at this SNP. n is the number of patients studied for each group. The horizontal lines in each plot represent the mean and the standard error of the mean. P-values were calculated using a Mann–Whitney *U* test for **A**), **B**) and **D**) and a Kruskal-Wallis test for **C**).

To initially assess the degree to which rs2615977 and rs1676486 correlate with expression of *COL11A1*, we used quantitative real-time PCR to measure the level of expression of the gene in cDNA that had been synthesised from cartilage RNA. We then stratified the data by genotype at rs2615977 and by genotype at rs1676486 (Figure 1). Only one patient was homozygous for the minor allele of rs1676486. This is a reflection of the low frequency of TT homozygotes for this SNP, with a frequency of only 1.8% reported in HapMap CEU. As such, for this SNP a comparison was made between those patients who carry the LDH risk T-allele and those who do not, that is, between CC homozygotes versus CT heterozygotes and TT homozygotes combined. There was no significant correlation (p < 0.05) between the level of *COL11A1* expression and the genotype at either rs2615977 or rs1676486. We subsequently stratified the patients into those who had undergone TKR and those who had undergone THR, and repeated the analysis. Again, there was no correlation between *COL11A1* expression and genotype at either SNP (Figure 2).

### AEI analysis

The above analysis did not provide evidence for a correlation between *COL11A1* expression and genotype at rs2615977 or genotype at rs1676486. However, this technique is vulnerable to the natural fluctuation in gene expression, which can decrease its sensitivity and accuracy [16]. For example, it is apparent from Figures 1 and 2 that between individuals of the same genotype the expression of *COL11A1* can vary by greater than 200 fold. As such, quantitative PCR can generate false negative results when used in this manner. An alternative method for correlating genotype with expression levels that is not vulnerable to such fluctuation is allelic expression imbalance, abbreviated to AEI and also known as differential allelic expression. This entails using a transcript SNP to differentiate and measure the cDNA output from the two alleles of a heterozygote. Genomic DNA is used as the comptor control to provide the 1:1 ratio to which the cDNA allelic ratio is compared. We therefore assessed whether *COL11A1* demonstrated AEI and if so, whether this correlated with genotype at rs2615977 or at rs1676486.

rs1676486 is a transcript SNP and it could itself be used in the AEI analysis. However, rs2615977 is intronic and could not be used. A search of the Ensembl database (http://www.ensembl.org/) for *COL11A1* transcript SNPs in high LD (r^2^ > 0.5) with rs2615977 identified only two SNPs, which happen to be in perfect LD (r^2^ = 1, D^′^ = 1) with the SNP: rs1012281 and rs1012282, both within exon 45. However, according to the UCSC database (http://genome.ucsc.edu/), the sequence harbouring these SNPs is not part of the *COL11A1* transcript. To resolve this discrepancy we amplified across the region using OA cartilage cDNAs and genomic DNAs as templates. The same primers were used for the two templates and we successfully amplified DNA but we failed to detect a cDNA product (results not shown). We concluded therefore that the UCSC database was accurate and that rs1012281 and rs1012282 are not transcript SNPs and could not therefore be used. Since there are no transcript SNPs in high LD with rs2615977 we opted to use a transcript SNP that had a high heterozygosity value, thus ensuring that a large number of our patients would be informative for the AEI analysis if we focused on individuals who were compound heterozygotes for rs2615977 and the transcript SNP. Due to the small size of the majority of *COL11A1* exons we needed to avoid a transcript SNP that was close to an intron/exon boundary, as this would not allow us to use the same primers and probe when targeting cDNA and DNA in our AEI analysis. The transcript SNP that we chose was the 3^′^UTR SNP rs9659030, which has a heterozygosity value of 0.31 in Europeans and is over 350 bp from the nearest exon boundary. Although rs9659030 is not in LD with rs2615977 (pair wise r^2^ = 0.08, pair wise D^′^ = 0.40), by studying compound heterozygotes we were able to use rs9659030 to functionally septe the two rs2615977 alleles. Although we could not determine phase between rs2615977 and rs9659030 we would expect a high proportion of the compound heterozygotes to demonstrate AEI if rs2615977 is itself, or if it correlates with, a polymorphism that influences gene expression at an allelic level.

#### rs1676486

**Figure 3:**
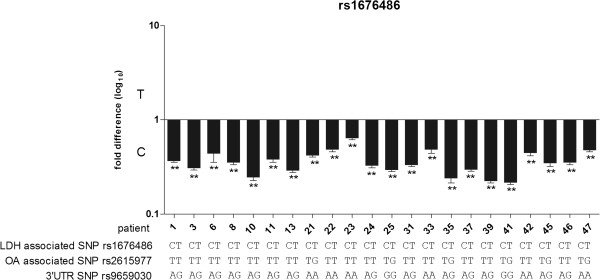
**Allelic expression analysis in cartilage samples from OA patients assessed using the LDH associated SNP rs1676486.** The genotypes of the patients at rs1676486, at the OA associated SNP rs2615977 and at the 3^′^UTR SNP rs9659030 are shown. P-values were calculated by comparing the cDNA allelic ratio to the DNA allelic ratio using a Mann–Whitney *U* test. ** denotes p < 0.01.

We carried out AEI analysis on 22 OA patients heterozygous for the LDH SNP rs1676486, all of whom showed a significant (p < 0.05) decrease in expression of the T-allele relative to the C-allele with an average allelic ratio of 0.36 (p < 0.0001) (Figure 3 and Additional file 5: Table S5). That is, for every one C-allele cDNA molecule there were only 0.36 cDNA molecules of a T-allele. It was the T-allele of rs1676486 that showed reduced stability in the original LDH study [9]. Our functional data therefore supported the functional data from the LDH study.

#### rs9659030

**Figure 4:**
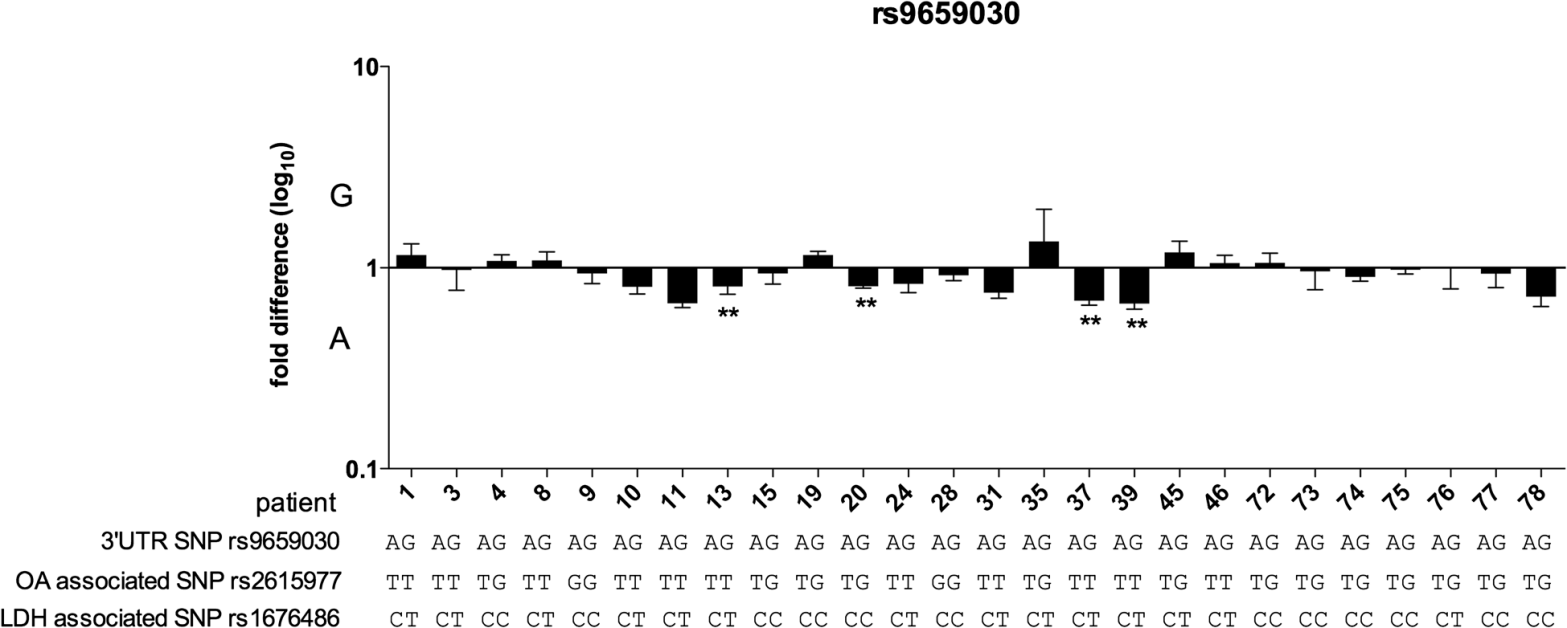
**Allelic expression analysis in cartilage samples from OA patients assessed using the 3**^**′**^**UTR SNP rs9659030.** The genotypes of the patients at rs9659030, at the OA associated SNP rs2615977 and at the LDH associated SNP rs1676486 are shown. P-values were calculated by comparing the cDNA allelic ratio to the DNA allelic ratio using a Mann–Whitney *U* test. ** denotes p < 0.01.

We carried out AEI analysis on 26 OA patients heterozygous for rs9659030 (Figure 4 and Additional file 5: Table S5). Thirteen of the 26 were compound heterozygous for rs2615977 and rs9659030 but only one of the 13, patient 20, showed a significant AEI. The two alleles of the OA associated SNP rs2615977 do not therefore correlate with differences in expression of *COL11A1*. In addition to patient 20 three other patients also showed a significant AEI (patients 13, 37 and 39 in Figure 4). These three patients were not heterozygous at rs2615977 but they were heterozygous for the LDH SNP rs1676486 and it is presumably this that accounts for the AEI observed in these patients.

We were intrigued to note that the further nine patients who were also compound heterozygous for rs1676486 and rs9659030 did not demonstrate significant AEI when their allelic expression was measured using rs9659030 (patients 1, 3, 8, 10, 11 24, 31, 46 and 76 in Figure 4) - based on the AEI results for rs1676486 reported earlier we had anticipated that all nine would have demonstrated significant AEI with rs9659030, particularly in light of the fact that eight of the nine had been tested with rs1676486 and had shown significant AEI with this SNP (patients 1, 3, 8, 10, 11, 24, 31 and 46 in Figure 3). Quality control (QC) checks on the rs9659030 AEI assay revealed that it is able to accurately discriminate between and measure differences in the levels of the two alleles of this SNP (Additional file 6: Figure S1) and that this capacity is well within the range of AEI observed at rs1676486. For example, the average rs1676486 C/T ratio for the 22 patients of Figure 3 and Additional file 5: Table S5 was 0.36 and it is clear from Additional file 6: Figure S1A and S1B that the rs9659030 AEI assay would have detected such a difference if it existed at this SNP. These QC checks confirm that the rs9659030 AEI assay is robust. Overall, these results imply that there is variation in the degradation rate of the *COL11A1* transcript across its length, which would mean that the AEI results obtained could partly be dependent on the physical position in the transcript of the SNP used to measure AEI.

### Genetic association analysis of rs1676486 with OA

We finally assessed whether rs1676486 was associated to OA using the arcOGEN GWAS dataset. This revealed that the SNP showed only marginal evidence of association, with a p-value of 0.012 in the comparison of 7,410 cases and 11,009 controls (Additional file 7: Table S6). The association signal was in the same direction as seen for the Japanese LDH study, in that it was the T-allele of rs1676486 that was elevated in cases (frequency of 0.201) versus controls (frequency of 0.1905) with an odds ratio of 1.069 (95% confidence interval of 1.015-1.127). Stratification of the arcOGEN data by site of OA (hip or knee) did not enhance the association signal (Additional file 7: Table S6) nor was there a more significant signal when the data was stratified by sex, or by sex combined with site (data not shown).

## Discussion

Allelic expression imbalance (AEI) has been shown to be an important mechanism through which OA susceptibility acts, with an excellent example being the SNP rs143383 located in the 5^′^UTR of the growth differentiation factor 5 gene *GDF5*; this SNP is robustly associated with knee OA across various ethnic groups, with the OA-associated T-allele of the polymorphism mediating reduced expression of *GDF5* in cartilage and other joint tissues [reviewed in 2]. Based on observations such as this, we were keen to assess whether the *COL11A1* OA association, marked by rs2615977, also correlated with AEI in OA cartilage. A previously published study of LDH demonstrated that this gene was subject to AEI and we therefore also assessed whether the LDH SNP rs1676486 correlated with AEI in OA cartilage and if so, whether the polymorphism was genetically associated with the disease.

Our results did not provide evidence for a correlation between the OA associated SNP rs2615977 and *COL11A1* AEI but they did provide compelling evidence for a correlation between the LDH associated SNP rs1676486 and AEI at this gene. As for LDH, it was the T-allele of rs1676486 that correlated with reduced expression of *COL11A1*. However, the polymorphism demonstrated only very weak evidence of association with OA and as this association data derives from a GWAS of over 480,000 SNPs, the very moderate p-value does not, in our view, support rs1676486 as an OA susceptibility locus despite its functional effect. From our rs1676486 data we therefore conclude that in the cartilage of OA patients who have undergone elective hip or knee joint replacement surgery, *COL11A1* is subject to AEI, but that this is not a risk factor for OA. Mature hyaline cartilage tissue is therefore resilient to such *COL11A1* allelic imbalance effects, unlike the fibrocartilage of LDH, which is vulnerable to *COL11A1* AEI.

The lack of a correlation between the OA associated SNP rs2615977 and *COL11A1* expression implies that the association marked by this SNP is operating by a route other than an effect on the expression of the gene in mature cartilage. However, it is possible that we failed to detect any such correlation, since in our AEI analysis we used a SNP, rs9659030, that is not physically close to rs2615977. After all, our comparison of AEI in individuals compound heterozygous for rs9659030 and rs1676486 did imply that AEI may not be uniform across the *COL11A1* transcript. However, the fact that AEI in mature cartilage as measured with rs1676486 is not an OA risk factor makes it seem unlikely that the OA risk marked by rs2615977 would be operating via this mechanism in this tissue. It is possible that rs2615977, or a SNP in high LD with it, is modulating the expression of *COL11A1* at time points not studied here, for example during skeletogenesis or in adolescent tissues. Of possible relevance to this is the presence in the ENCODE database [17] of SNP rs11164648, which is in perfect LD with rs2615977 and which is located within a CTCF-binding site that is predicted to act as a transcriptional insulator. CTCF is a transcriptional repressor and CTCF-dependent insulators have been shown to regulate gene expression by modulating chromatin conformation during development [18]. If cartilage tissues were to become available from an adequate number of young donors then assessing *COL11A1* AEI relative to genotype at rs2615977 or rs11164648 may be insightful.

As noted in the introduction, Stickler syndrome is a rare and usually dominant condition presenting with a severe and early onset form of OA [7,8]. Stickler syndrome can present with a high degree of inter and intra-familial variability and as such it may be of benefit to assess whether the possession of the low expressing T-allele of rs1676486, which we have shown is functionally active in cartilage and which has a frequency of >20% in Europeans and Asians, correlates in any way with this phenotypic heterogeneity; possessing a low expressing *COL11A1* allele alongside a mutant allele may exacerbate the phenotype whilst if the mutant *COL11A1* allele is on a low-expressing allele then this could attenuate the phenotype.

## Conclusions

Overall our study has identified that *COL11A1* AEI is common to both LDH and OA, but that it is not a risk factor for OA. Presumably other functional effects must be accounting for the OA associated signal at this gene.

## Competing interests

The authors declare that they have no competing interests.

## Authors’ contributions

All authors were involved in drafting the article or revising it critically for important intellectual content, and all authors approved the final version to be published. Prof. L had full access to all of the data in the study and takes responsibility for the integrity of the data and the accuracy of the data analysis. Study conception and design. D, R, L. Acquisition of data. R, D. Analysis and interpretation of data. R, D, R, L.

## Authors’ information

Emma V A Raine and Andrew W Dodd: The authors wish it to be known that, in their opinion, the first two authors should be regarded as joint first authors.

## Pre-publication history

The pre-publication history for this paper can be accessed here:

http://www.biomedcentral.com/1471-2474/14/85/prepub

## Supplementary Material

Additional file 1: Table S1 Table of patient characteristics and their genotype at the three *COL11A1* SNPs studied in this report. F, female; M, male, K, knee, H, hip. Click here for file

Additional file 2: Table S2 Summary table of the osteoarthritis (OA) patients studied. Click here for file

Additional file 3: Table S3 Table of primers and enzymes used for genotyping SNPs by RFLP analysis. Click here for file

Additional file 4: Table S4 Table of the PrimeTime Mini qPCR Assays for quantitative PCR analysis of *COL11A1*. Click here for file

Additional file 5: Table S5 Table of the allelic ratios for the patients analysed for AEI at rs9659030 and rs1676486. Allelic ratios are shown ± the standard error of the mean. P-values were calculated using a Mann Whitney *U* test. P-values < 0.05 are highlighted in bold. Click here for file

Additional file 6: Figure S1 Quality control of the rs9659030 allelic expression imbalance (AEI) assay. Click here for file

Additional file 7: Table S6 Association analysis of rs1676486 with OA using the arcOGEN data. Click here for file

## References

[B1] Brandt KD Dieppe P Radin E Etiopathogenesis of osteoarthritis Rheum Dis Clin North Am 2008 34 531 559 10.1016/j.rdc.2008.05.011 18687271

[B2] Loughlin J Genetics of osteoarthritis Curr Opin Rheumatol 2011 23 479 483 10.1097/BOR.0b013e3283493ff0 21709558

[B3] Kerkhof HJM Lories RJ Meulenbelt I Jonsdottir I Valdes AM Arp P Ingvarsson T Jhamai M Jonsson H Stolk L Thorleifsson G Zhai G Zhang F Zhu Y van der Breggen R Carr A Doherty M Doherty S Felson DT Gonzalez A Halldorsson BV Hart DJ Hauksson VB Hofman A Ioannidis JP Kloppenburg M Lane NE Loughlin J Luyten FP Nevitt MC A genome-wide association study identifies an osteoarthritis susceptibility locus on chromosome 7q22 Arthritis Rheum 2010 62 499 510 20112360 10.1002/art.27184 PMC3354739

[B4] Panoutsopoulou K Southam L Elliott KS Wrayner N Zhai G Beazley C Thorleifsson G Arden NK Carr A Chapman K Deloukas P Doherty M McCaskie A Ollier WE Ralston SH Spector TD Valdes AM Wallis GA Wilkinson JM Arden E Battley K Blackburn H Blanco FJ Bumpstead S Cupples LA Day-Williams AG Dixon K Doherty SA Esko T Evangelou E Insights into the genetic architecture of osteoarthritis from stage 1 of the arcOGEN study Ann Rheum Dis 2011 70 864 867 10.1136/ard.2010.141473 21177295 PMC3070286

[B5] Day-Williams AG Southam L Panoutsopoulou K Rayner NW Esko T Estrada K Halgadottir H Hofman A Ingvarsson T Jonsson H Keis A Kerkhof HJ Throleifsson G Arden NK Carr A Chapman K Deloukas P Loughlin J McCaskie A Ollier WE Ralston SH Spector TD Wallis GA Wilkinson JM Aslam N Birell F Carluke I Joseph J Rai A Reed M Walker K A variant in MCF2L is associated with osteoarthritis Am J Hum Genet 2011 89 446 450 10.1016/j.ajhg.2011.08.001 21871595 PMC3169824

[B6] arcOGEN Consortium and arcOGEN Collaborators Identification of new susceptibility loci for osteoarthritis (arcOGEN): a genome-wide association scan Lancet 2012 380 815 823 22763110 10.1016/S0140-6736(12)60681-3 PMC3443899

[B7] Kannu P Bateman JF Belluoccio D Fosang AJ Savarirayan R Employing molecular genetics of chondrodysplasias to inform the study of osteoarthritis Arthritis Rheum 2009 60 325 334 10.1002/art.24251 19180483

[B8] Richards AJ McNinch A Martin H Oakhill K Rai H Waller S Treacy B Whittaker J Meredith S Poulson A Snead MP Stickler syndrome and the vitreous phenotype: mutations in COL2A1 and COL11A1 Hum Mutat 2010 31 E1461 E1471 10.1002/humu.21257 20513134

[B9] Mio F Chiba K Hirose Y Kawaguchi Y Mikami Y Oya T Mori M Kamata M Matsumoto M Ozaki K Tanaka T Takahashi A Kubo T Kimura T Toyama Y Ikegawa S A functional polymorphism in COL11A1, which encodes the α1 chain of type XI collagen, is associated with susceptibility to lumbar disc herniation Am J Hum Genet 2007 81 1271 1277 10.1086/522377 17999364 PMC2276353

[B10] Cookson W Liang L Abecasis G Moffat M Lathrop M Mapping complex disease traits with global gene expression Nat Rev Genet 2009 10 184 194 10.1038/nrg2537 19223927 PMC4550035

[B11] Montgomery SB Dermitzakis ET From expression QTLs to personalized transriptomics Nat Rev Genet 2011 12 277 282 21386863 10.1038/nrg2969

[B12] Musunuru K Strong A Frank-Kamenetsky M Lee NE Ahfeldt T Sachs KV Li X Li H Kuperwasser N Ruda VM Pirruccello JP Muchmore B Prokunina-Olsson L Hail J Schadt EE Morales CR Lund-Katz S Phillips MC Wong J Cantley W Racie T Ejebe KG Orho-Melander M Melander O Koteliansky V Fitzgerald K Krauss RM Cowan CA Kathiresan S Rader DJ From noncoding variant to phenotype via SORT1 at the 1p13 cholesterol locus Nature 2010 466 714 719 10.1038/nature09266 20686566 PMC3062476

[B13] Fairfax BP Makino S Radhakrishnan J Plant K Leslie S Dilthey A Ellis P Langford C Vannberg FO Knight JC Genetics of gene expression in primary immune cells identifies cell type-specific master regulators and roles of HLA alleles Nature Genet 2012 44 502 510 10.1038/ng.2205 22446964 PMC3437404

[B14] Southam L Rodriguez-Lopez J Wilkins JM Pombo-Suarez M Snelling S Gomez-Reino JJ Chapman K Gonzalex A Loughlin J A SNP in the 5^′^UTR of GDF5 is associated with osteoarthritis susceptibility in Europeans and with in vivo differences in allelic expression in articular cartilage Hum Mol Genet 2007 16 2226 2232 10.1093/hmg/ddm174 17616513

[B15] Wilkins JM Southam L Price AJ Mustafa Z Carr A Loughlin J Extreme context specificity in differential allelic expression Hum Mol Genet 2007 16 537 546 10.1093/hmg/ddl488 17220169

[B16] Johnson AD Zhang Y Papp AC Pinsonneault JK Lim JE Saffen D Dai Z Wang D Sandee W Polymorphisms affecting gene transcription and mRNA processing in pharmacogenetic candidate genes: detection through allelic expression imbalance in human target tissues Pharmacogenet Genomics 2008 18 781 791 10.1097/FPC.0b013e3283050107 18698231 PMC2779843

[B17] Maher B ENCODE: The human encyclopaedia Nature 2012 489 46 48 10.1038/489046a 22962707

[B18] Furlan-Magaril M Rebollar E Guerrero G Fernandez A Molto E Gonzalez-Buendia E Cantero M Montoliu L Recillas-Targa F An insulator embedded in the chicken α-globin locus regulates chromatin domain configuration and differential gene expression Nucleic Acids Res 2011 39 89 103 10.1093/nar/gkq740 20813760 PMC3017597

