# Supplementary material for: Allelic expression analysis of the osteoarthritis susceptibility gene COL11A1 in human joint tissues
Source: BMC Musculoskelet Disord. 2013 Mar 8;14:85. doi: 10.1186/1471-2474-14-85 (PMC3599795; doi:10.1186/1471-2474-14-85)
Supplement: Additional file 1: Table S1 — Table of patient characteristics and their genotype at the three COL11A1 SNPs studied in this report. F, female; M, male, K, knee, H, hip. [file 1471-2474-14-85-S1.pdf]

Additional file 1: Table S1 Table of patient characteristics and their genotype at the three *COL11A1* SNPs studied in this report. F, female; M, male, K, knee, H, hip.

Patient	Sex	Age at surgery (years)	Joint replaced at surgery	Genotype		
				rs2615977	rs1676486	rs9659030
1	F	75	H	TT	CT	AG
2	F	67	K	TT	CC	AA
3	M	69	K	TT	CT	AG
4	F	77	H	TG	CC	AG
5	F	73	K	TT	CC	AA
6	M	71	K	TT	CT	AG
7	F	73	K	GG	CC	AA
8	F	51	H	TT	CT	AG
9	F	60	K	GG	CC	AG
10	F	67	K	TT	CT	AG
11	M	62	K	TT	CT	AG
12	M	50	K	TG	CC	AA
13	M	67	K	TT	CT	AG
14	F	76	H	TT	CC	AA
15	F	71	K	TG	CC	AG
16	F	67	H	TT	CC	AA
17	F	70	H	TT	CC	AA
18	F	60	H	TG	CC	AA
19	M	80	K	TG	CC	AG
20	F	81	K	TG	CC	AG
21	M	76	K	TG	CT	AA
22	F	55	K	TT	CT	AA
23	M	57	K	TT	CT	AA
24	M	57	H	TT	CT	AG
25	M	69	K	TG	CT	GG
26	F	67	K	TT	CC	AA
27	F	69	K	GG	CC	AA
28	M	57	K	GG	CC	AG
29	F	60	K	TG	CC	AA
30	F	66	K	TT	CC	AA
31	M	63	K	TT	CT	AG
32	M	77	K	TT	CC	AA
33	M	82	K	TT	CT	AA
34	F	78	K	TT	CC	AA
35	M	82	K	TG	CT	AG
36	M	46	K	TT	CC	AA
37	M	56	K	TT	CT	AG
38	F	54	K	TT	TT	GG
39	M	71	K	TT	CT	AG
40	F	58	H	TT	CC	AA
41	F	69	H	TG	CT	GG
42	F	64	K	TT	CT	AA

43	M	63	K	TG	CC	AA
44	F	71	H	TG	CC	AG
45	M	70	K	TG	CT	AG
46	M	67	K	TT	CT	AG
47	M	86	K	TT	CT	AA
48	F	67	K	TT	CC	AA
49	M	71	K	TG	CC	AA
50	F	46	K	TT	CC	AA
51	F	62	K	TT	CC	AA
52	F	58	K	TG	CC	AA
53	M	69	K	TG	CC	AG
54	M	59	K	TT	CT	AG
55	M	64	K	TT	CT	GG
56	F	81	K	GG	CC	AG
57	F	80	K	TT	CT	AG
58	F	64	K	TG	CC	AA
59	F	78	K	TG	CC	AG
60	F	61	K	TT	CT	AG
61	F	80	K	TG	CT	GG
62	F	80	K	TT	CC	AA
63	F	59	K	TG	CT	AG
64	F	71	H	TT	CT	AG
65	M	74	K	GG	CC	AG
66	F	74	H	TT	CC	AG
67	M	72	K	TG	CC	AG
68	M	72	K	TT	CC	AA
69	M	68	H	TG	CC	AA
70	F	72	H	TG	CC	AA
71	M	68	K	TG	CC	AA
72	M	75	K	TG	CC	AG
73	F	82	H	TG	CC	AG
74	F	78	K	TG	CC	AG
75	M	72	K	TG	CC	AG
76	F	59	K	TG	CC	AG
77	F	71	H	TG	CC	AG
78	M	69	K	TG	CC	AG
