# Supplementary material for: Allelic expression analysis of the osteoarthritis susceptibility gene COL11A1 in human joint tissues
Source: BMC Musculoskelet Disord. 2013 Mar 8;14:85. doi: 10.1186/1471-2474-14-85 (PMC3599795; doi:10.1186/1471-2474-14-85)
Supplement: Additional file 2: Table S2 — Summary table of the osteoarthritis (OA) patients studied. [file 1471-2474-14-85-S2.pdf]

Additional file 2: Table S2. Summary table of the osteoarthritis (OA) patients studied

Number	% Female	Mean age at surgery (age range), years		Strata (number)			
		Female	Male	Female-hip	Female-knee	Male-hip	Male-knee
78	56	68 (46-82)	68 (46-86)	15	29	2	32
