# Supplementary material for: Allelic expression analysis of the osteoarthritis susceptibility gene COL11A1 in human joint tissues
Source: BMC Musculoskelet Disord. 2013 Mar 8;14:85. doi: 10.1186/1471-2474-14-85 (PMC3599795; doi:10.1186/1471-2474-14-85)
Supplement: Additional file 3: Table S3 — Table of primers and enzymes used for genotyping SNPs by RFLP analysis. [file 1471-2474-14-85-S3.pdf]

Additional file 3: Table S3. Table of primers and enzymes used for genotyping SNPs by RFLP analysis

SNP	Forward primer (5'-3')	Reverse primer (5'-3')	Enzyme
rs2615977	GTTTTAAAACATCCCCAGATAATTATCATATTCACTG	GTAACTAACCTGCACGTTGT	BsrI
rs1676486	GCATTTTGTAGGGTCCTCAAGGC	ACTTTGCTATGGTAGCCCTAGC	BsaI
rs9659030	GACCTACCTAATTGCTAAATGAATAACATATGGTGGACTGTTATTAACAG	ACTGTTCCAGTGAAATCTGG	TspRI
