# Supplementary material for: Allelic expression analysis of the osteoarthritis susceptibility gene COL11A1 in human joint tissues
Source: BMC Musculoskelet Disord. 2013 Mar 8;14:85. doi: 10.1186/1471-2474-14-85 (PMC3599795; doi:10.1186/1471-2474-14-85)
Supplement: Additional file 4: Table S4 — Table of the PrimeTime Mini qPCR Assays for quantitative PCR analysis of COL11A1. [file 1471-2474-14-85-S4.pdf]

Additional file 4: Table S4. Table of the PrimeTime Mini qPCR Assays for quantitative PCR analysis of *COL11A1*

Gene	Primer 1 (5'-3')	Primer 2 (5'-3')	Probe
COL11A1	TTCTCCACGCTGATTGCTAC	TTGGTGTTGAGGTTGGGAG	56-FAM/TTAACATCGCTGACGGGAAGTGGC/36-TAMSp
HPRT1	TGCTGAGGATTTGGAAAGGG	ACAGAGGGCTACAATGTGATG	56-FAM/AGGACTGAACGTCTTGCTCGAGATG/36-TAMSp
GAPDH	GGCCATCCACAGTCTTCTG	CAGCCTCAAGATCATCAGCAA	56-FAM/ATGACCACA/ZEN/GTCCATGCCATCACT/31ABkFQ
18S	CGAATGGCTCATTAAATCAGTTATGG	TATTAGCTCTAGAATTACCACAGTTATCC	56-FAM/TCCTTTGGTCGCTCGCTCCTCTCCC/TAMRA
