# Supplementary material for: Allelic expression analysis of the osteoarthritis susceptibility gene COL11A1 in human joint tissues
Source: BMC Musculoskelet Disord. 2013 Mar 8;14:85. doi: 10.1186/1471-2474-14-85 (PMC3599795; doi:10.1186/1471-2474-14-85)
Supplement: Additional file 5: Table S5 — Table of the allelic ratios for the patients analysed for AEI at rs9659030 and rs1676486. Allelic ratios are shown ± the standard error of the mean. P-values were calculated using a Mann Whitney U test. P-values < 0.05 are highlighted in bold. [file 1471-2474-14-85-S5.pdf]

Additional file 5: Table S5. Table of the allelic ratios for the patients analysed for AEI at rs9659030 and rs1676486. Allelic ratios are shown ± the standard error of the mean. P-values were calculated using a Mann Whitney U test. P-values < 0.05 are highlighted in bold.

rs9659030			rs1676486		
Patient	Allelic ratio (A/G)	P value	Patient	Allelic ratio (C/T)	P value
1	1.16 ± 0.16	0.42	**1**	**0.36 ± 0.01**	**<0.01**
3	0.98 ± 0.2	1.00	**3**	**0.31 ± 0.01**	**<0.01**
4	1.08 ± 0.08	0.84	**6**	**0.43 ± 0.08**	**<0.01**
8	1.09 ± 0.11	0.55	**8**	**0.35 ± 0.02**	**<0.01**
9	0.94 ± 0.1	1.00	**10**	**0.24 ± 0.02**	**<0.01**
10	0.8 ± 0.06	0.1	**11**	**0.38 ± 0.02**	**<0.01**
**11**	**0.66 ± 0.03**	**<0.01**	**13**	**0.29 ± 0.01**	**<0.01**
13	0.81 ± 0.07	0.06	**21**	**0.42 ± 0.01**	**<0.01**
15	0.94 ± 0.12	0.69	**22**	**0.48 ± 0.02**	**<0.01**
19	1.16 ± 0.05	0.06	**23**	**0.64 ± 0.02**	**<0.01**
**20**	**0.81 ± 0.02**	**<0.01**	**24**	**0.33 ± 0.02**	**<0.01**
24	0.83 ± 0.08	0.22	**25**	**0.29 ± 0.01**	**<0.01**
28	0.92 ± 0.05	0.55	**31**	**0.33 ± 0.01**	**<0.01**
31	0.75 ± 0.05	0.15	**33**	**0.48 ± 0.04**	**<0.01**
35	1.35 ± 0.6	0.33	**35**	**0.24 ± 0.03**	**<0.01**
**37**	**0.69 ± 0.03**	**<0.01**	**37**	**0.29 ± 0.01**	**<0.01**
**39**	**0.66 ± 0.04**	**<0.01**	**39**	**0.22 ± 0.01**	**<0.01**
45	1.2 ± 0.16	0.69	**41**	**0.22 ± 0.01**	**<0.01**
46	1.06 ± 0.1	0.69	**42**	**0.44 ± 0.03**	**<0.01**
72	1.06 ± 0.12	0.73	**45**	**0.34 ± 0.03**	**<0.01**
73	0.96 ± 0.18	0.57	**46**	**0.35 ± 0.02**	**<0.01**
74	0.90 ± 0.04	1.00	**47**	**0.47 ± 0.02**	**<0.01**
75	0.98 ± 0.04	1.00			
76	0.99 ± 0.21	0.79			
77	0.93 ± 0.14	0.69			
78	0.72 ± 0.08	0.55			
