# Supplementary material for: Allelic expression analysis of the osteoarthritis susceptibility gene COL11A1 in human joint tissues
Source: BMC Musculoskelet Disord. 2013 Mar 8;14:85. doi: 10.1186/1471-2474-14-85 (PMC3599795; doi:10.1186/1471-2474-14-85)
Supplement: Additional file 6: Figure S1 — Quality control of the rs9659030 allelic expression imbalance (AEI) assay. [file 1471-2474-14-85-S6.pdf]

A

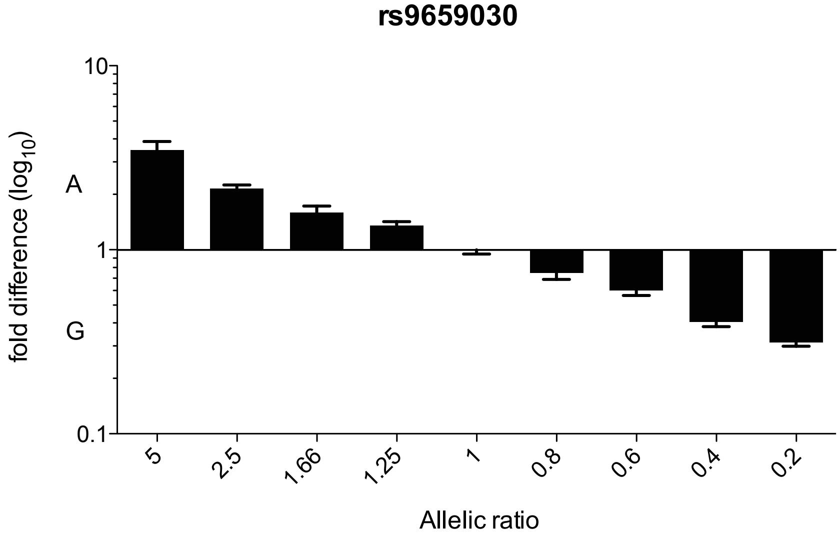

B

	Allelic ratios								
Expected	5	2.5	1.66	1.25	1	0.8	0.6	0.4	0.2
Observed	3.5	2.1	1.6	1.4	1	0.8	0.6	0.4	0.3

C

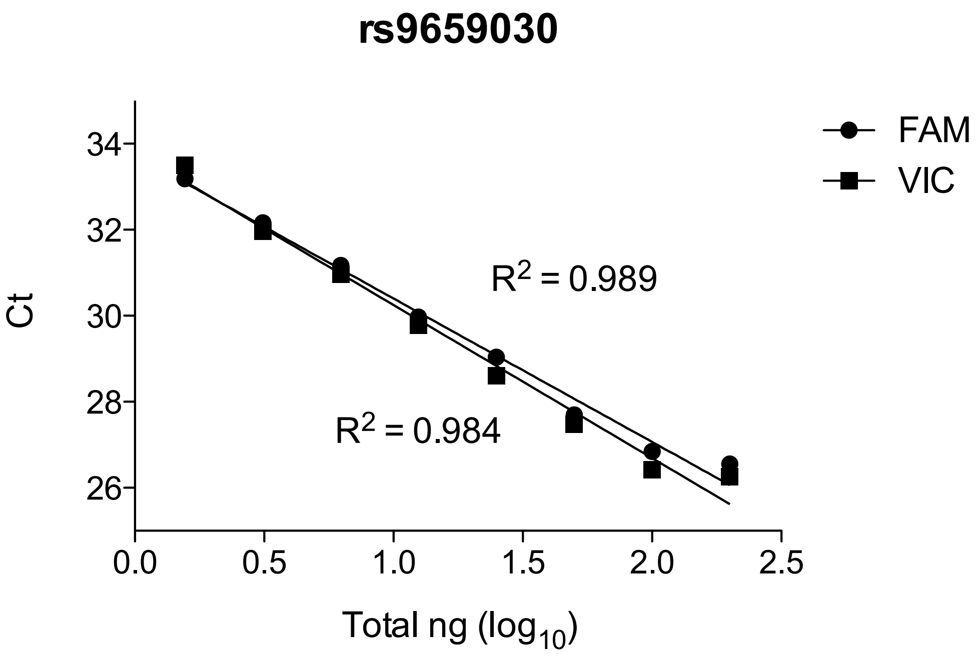

Additional file 6: Figure S1. Quality control of the rs9659030 allelic expression imbalance (AEI) assay. A) Allelic ratios ranging from 5:1 to 1:1 in both allelic directions were prepared by mixing DNAs homozygous AA and homozygous GG at rs9659030. AEI was then performed on each sample, with three technical repeats. The error bars are the standard error of the mean. The x-axis lists the 9 ratios tested. B) Table showing the ratios tested (expected) and their respective values as measured by the AEI assay (observed). C) Serial dilution of a heterozygous DNA sample to test for the amplification efficiencies of the A (FAM) and G (VIC) probes of the rs9659030 AEI assay. The dilution range was from 200ng to 1.5635ng of DNA and is represented on the x-axis as $\log_{10}$. The cycle threshold (Ct) values are represented on the y-axis. R^2 is the coefficient of determination.
