# Supplementary material for: Allelic expression analysis of the osteoarthritis susceptibility gene COL11A1 in human joint tissues
Source: BMC Musculoskelet Disord. 2013 Mar 8;14:85. doi: 10.1186/1471-2474-14-85 (PMC3599795; doi:10.1186/1471-2474-14-85)
Supplement: Additional file 7: Table S6 — Association analysis of rs1676486 with OA using the arcOGEN data. [file 1471-2474-14-85-S7.pdf]

Additional file 7: Table S6. Association analysis of rs1676486 with OA using the arcOGEN data

Stratum	Number of cases	Number of controls	T-allele frequency		P-value	Odds ratio (95% confidence intervals)
			Cases	Controls		
All cases	7410	11009	0.201	0.1905	0.012	1.069 (1.015-1.127)
Hip cases	3912	11009	0.201	0.1905	0.043	1.069 (1.002-1.141)
Knee cases	4144	11009	0.2035	0.1905	0.010	1.086 (1.02-1.157)
